# Association of the Triglyceride Glucose Index With Outcomes in Acute Ischemic Stroke Injury

**DOI:** 10.7759/cureus.75841

**Published:** 2024-12-16

**Authors:** Rishita Garg, Mini Bhatnagar, Sunita Gupta

**Affiliations:** 1 General Medicine, Maharishi Markandeshwar Institute of Medical Sciences and Research, Mullana, IND

**Keywords:** ais, neurological worsening, outcomes, stroke prognosis, triglyceride glucose index

## Abstract

Objectives

The study aimed at estimating the triglyceride glucose (TyG) index in patients with ischemic stroke, exploring the correlation between the TyG index and the prognosis of ischemic stroke, and studying the clinical outcome in patients with acute ischemic stroke (AIS) in association with the TyG index.

Methods

An observational study was conducted on 105 patients with a history of AIS presenting within 24 hours. The TyG index was estimated, and the clinical outcome was studied. The outcome measures were neurological worsening (National Institutes of Health Stroke Scale (NIHSS) >=2 gain at discharge above admission), poor functional outcomes (assessed by derangement of modified Rankin scale (mRS) at three months), stroke recurrence, and three-month mortality rate. The NIHSS and mRS were assessed at admission, discharge, and three months later.

Results

The mean TyG index of AIS patients was 9.26±0.2. Compared to patients without neurological worsening (n=8), patients with neurological worsening had a significantly higher TyG index (9.5±0.19 vs. 9.21±0.21, p<.0001). Compared to patients without poor functional outcomes (n=20), patients with poor functional outcomes had a similar TyG index (9.31±0.31 vs. 9.25±0.22, p=0.418). Compared to patients without recurrence, those with recurrence of stroke had a significantly higher TyG index (9.55±0.18 vs. 9.15±0.15, p<.0001). On the follow-up of three months post-stroke, compared to survivors, non-survivors had a similar TyG index (9.12±0.25 vs. 9.27±0.24, p=0.09).

Conclusion

The TyG index showed a significant association with outcomes of AIS in terms of stroke recurrence and neurological worsening. Overall, it shows that insulin resistance has a significant impact on the neurological outcomes and recurrence of stroke in AIS patients.

## Introduction

Stroke is defined by a local or global interference in the brain’s activity that is vascular in nature and lasts for more than 24 hours [1]. Recent data taken from the National Stroke Registry Programme, India, suggests that stroke’s pooled crude incidence rate is 138.1 per 100,000 population [2].

When there is further narrowing of arteries, due to a thrombi or emboli, in a patient having atherosclerosis, it leads to ischemic stroke as the cerebral blood flow decreases. It deteriorates the demand-supply ratio of oxygen and blood, causing a permanent ischemic zone with a hypoperfused zone of penumbra on the periphery [3]. Among hemorrhagic stroke and Ischemic stroke, the occurrence rate of ischemic stroke is 75% to 80% [1].

Conditions like hypertension, overweight, diabetes mellitus, smoking, old age, alcohol, obesity, ethnicity, and cardiac diseases, which can lead to blockage of blood vessels or plaque formation, are categorized as risk factors [2].

Among all the factors leading to stroke, insulin resistance (IR) is the main culprit [4]. As per research, IR leads to platelet aggregation disorders, hemodynamic disturbances, and atherosclerosis. All these factors in turn lead to stroke and also increase the likelihood of reappearance [5-8].

In clinical settings, IR is checked most frequently with the Homeostatic Model Assessment for Insulin Resistance (HOMA-IR), even though it has lower practical application. The triglyceride glucose (TyG) index is a newer way of checking IR and it is calculated as follows:

ln [fasting triglycerides (mg/dl)×fasting blood glucose (mg/dl)/2] [9]. Below 8 is the normal cut-off range of the TyG index to be considered normal [10].

This newer index is better than the previously used indexes, like HOMA-IR, as it is uncomplicated, making it easy on the pocket and favorable for patients at the initial stages. It is simple in nature because it is a mathematical model that analyzes fasting plasma glucose and triglyceride to gauge IR. The TyG index is a proxy marker for IR assessment. Also, it promises a superior accuracy with specificity values in the range of 32.5% to 85%, and sensitivity values in the range of 67% to 96% [11].

To validate the TyG index, many research studies were used to make a comparison of the TyG index with HOMA-IR and the hyperinsulinemic-euglycemic clamp test [12-14]. Prior research suggests a connection of the TyG index with coronary artery calcification, carotid atherosclerosis, increased risk of cardiovascular disease, and coronary artery stenosis; however, data to support the TyG index having substandard results in ischemic stroke patients is scarce [15-22].

Therefore, this study was performed to find out the association between clinical outcomes in acute ischemic stroke (AIS) patients and the TyG index, so that it can be used in the future as a clinical indicator for prognosis.

## Materials and methods

The study was done in the Department of Medicine, MMIMSR, Mullana (tertiary care hospital). The study design was observational; the study was conducted on a sample size of 105 AIS patients coming to the hospital for a year (Mar 23-Apr 24).

The inclusion criteria for the study were patients with a history of AIS presenting to the hospital within 24 hours, as per the World Health Organization-defined stroke criteria. Participants must be older than 18 years and have valid brain imaging reports, such as computed tomography (CT) or magnetic resonance imaging (MRI), confirming the diagnosis. Additionally, only those who have signed the informed consent form will be included. On the other hand, the exclusion criteria included patients with trauma, hemorrhagic stroke, or associated comorbidities, including hepatic or renal failure, cancer, and post-operative conditions.

After explaining to patients about the study, an informed consent form is obtained. Approval of ethical clearance is obtained (IEC- 2530). Post that a detailed medical history is taken and a physical examination is performed. To provide a thorough knowledge of the study to patients, a patient information sheet is given to them. To establish the diagnosis of acute stroke, the following data was taken: temporal profile of the clinical symptoms, clinical examination, and brain CT or MRI.

As soon as the patients were admitted, blood samples were taken in fasting (patients were asked to fast for a minimum of eight hours during the night) to measure lipid and glucose levels using an automated enzymatic approach. To calculate the TyG index following formula was used: ln [fasting triglycerides (mg/dl) × fasting glucose (mg/ dl)/2] [23].

Blood samples were collected from the antecubital vein. BD Vacutainer tubes were used to keep the samples mixed with EDTA, i.e. ethylenediamine tetraacetic acid. A centrifuge machine was used then to separate serum from the whole blood. A fully automated biochemistry auto-analyzer (Cobas 6000 biochemistry analyzer, Roche Diagnostics, Switzerland, Europe) was used to measure Fasting plasma glucose (FPG), triglycerides, total cholesterol, serum low-density lipoprotein cholesterol, and serum high-density lipoprotein cholesterol.

Outcomes were noted down as discharged or died. Patients were monitored on their extent of neurological deterioration post-stroke during their time in the hospital. An increase of 2 or more points in the National Institutes of Health Stroke Scale (NIHSS) score during their stay in hospital in comparison to the same score during admission was used to define neurological deterioration [24]. The modified Rankin Scale (mRS) was used to note the functional outcomes. Assessment of the mRS and NIHSS was done at three stages: admission, discharge, and three months.

Post-discharge, monitoring of patients was done for three months for the following clinical outcomes: (a) neurological deterioration (defined as an increase in NIHSS score by 2 or more points), (b) recurrent stroke (including intracranial hemorrhage, ischemic stroke, and subarachnoid hemorrhage), (c) all-cause mortality (tracked by telephonic contact), (d) mRS score (poor functional outcome being mRS score of 3-6 after three months). Correlation of all these outcomes (taken after three months) was done with the TyG Index (taken at the time of admission).

Microsoft Office Excel (Microsoft Corporation, Redmond, USA) was used to feed required patient data into the computer for statistics. The patient data was put into two subsets of variables i.e. quantitative or non-categorical and qualitative or categorical. Percentage and number are used to show categorical variables. Calculations were made of median values and interquartile range (25th to 75th percentiles). Table 1 categorizes the various statistical tests used for various comparisons.

**Table 1:** Standard tests and measures NIHSS: National Institutes of Health Stroke Scale; mRS: modified Rankin Scale; TyG: triglyceride glucose

Test name	Use
Shapiro-Wilk test	data distribution (normal vs non-normal)
Independent t-test	TyG index relation with results
Wilcoxon signed-rank test	To compare NIHSS scores across follow-up
Paired t-test	To compare the modified Rankin scale across follow-up
Spearman rank correlation coefficient	To assess the correlation between the TyG index and NIHSS, mRS in the follow-up
Statistical Package for Social Sciences (SPSS) software (version 25) manufactured by IBM, Chicago, USA	To analyze the whole subset of data taking P less than 0.05 as a cut-off point for statistical significance

## Results

The total number of participants enrolled in the study was 105. Male patients accounted for 66 (62.86%) and female patients accounted for 39 (37.14%), with a mean age of 69.71 years (standard deviation 12.1 years). Table 2 illustrates the demographics of patients.

**Table 2:** Demographic characteristics of AIS patients BMI: Body mass index; CHD: congenital heart disease; OSA: obstructive sleep apnea; RHD: rheumatic heart disease; AIS: acute ischemic stroke

Parameters	Mean+-SD	N (%)
Age (years)	69.71 ± 12.1	
Gender		
Male		66 (62.86%)
Females		39 (37.14%)
Presenting complaints		
Hemiparesis		64 (60.95%)
Aphasia		24 (22.86%)
Slurred speech		22(20.95%)
Vertigo		20 (19.05%)
Headache		15 (14.29%)
Facial deviation		15 (14.29%)
Cerebellar symptoms		9 (8.57%)
Ataxia		6 (5.71%)
Seizures		5 (4.76%)
Upper limb weakness		5 (4.76%)
Facial weakness		5 (4.76%)
Drowsiness		3 (2.86%)
Unresponsiveness		2 (1.90%)
Facial palsy		2 (1.90%)
Dizziness		2 (1.90%)
Altered sensorium		2 (1.90%)
Decreased responsiveness		1 (0.95%)
Addictions		
Smokers		13 (12.38%)
Alcoholic		17 (16.19%)
Comorbidities		
Hypertension		82 (78.10%)
Diabetes mellitus		76 (72.38%)
CHD		48 (45.71%)
RHD		1 (0.95%)
OSA		1 (0.95%)
Past history of stroke		10 (9.52%)
Family history of stroke		19 (18.10%)
BMI (in kg/m2)	24.34 ± 3.26	

At the time of admission, vitals were taken. Mean values of vitals are as follows: temperature 98.78 ± 0.89°F, pulse rate 85.98 ± 17.29 per minute, systolic blood pressure 150.62±18.17 mmHg, diastolic blood pressure 81.85±8.63 mmHg, and respiratory rate 21.2±3.64 per minute. Table 3 illustrates investigations performed.

**Table 3:** Investigations HDL: High-density lipoprotein; LDL: low-density lipoprotein; VLDL: very-low-density lipoprotein; ESR: erythrocyte sedimentation rate

Tests	Mean+-SD
Hemoglobin(g/dL)	11.82 ± 1.59
Total leucocyte count(cells/mm³)	10350.48 ± 4863.92
Platelet count(cells/mm³)	2.65 ± 0.68
ESR(mm/hour)	44.52 ± 2.59
Fasting blood glucose(mg/dL)	144.63 ± 23.04
Urea(mg/dL)	32.07 ± 19.57
Creatinine(mg/dL)	0.83 ± 0.47
Lipid profile	
HDL(mg/dL)	40.73 ± 3.13
LDL(mg/dL)	100.96 ± 10.53
VLDL(mg/dL)	29.68 ± 3.86
Triglyceride(mg/dL)	148.38 ± 19.31
Total cholesterol(mg/dL)	171.37 ± 11

The TyG index mean value among the subjects taken in the study was 9.26 ± 0.2. The TyG median index was 9.2(9.08-9.4) along with the 25th to 75th percentile range. The cut-off value for the TyG index was 8; however, all the patients had a higher TyG index value (Table 4).

**Table 4:** Descriptive statistics of the TyG index TyG: Triglyceride glucose

Variable	Mean ± SD	Median(25th-75th percentile)	Range
TyG index	9.26 ± 0.2	9.2(9.08-9.4)	8.88-9.88

**Figure 1:**
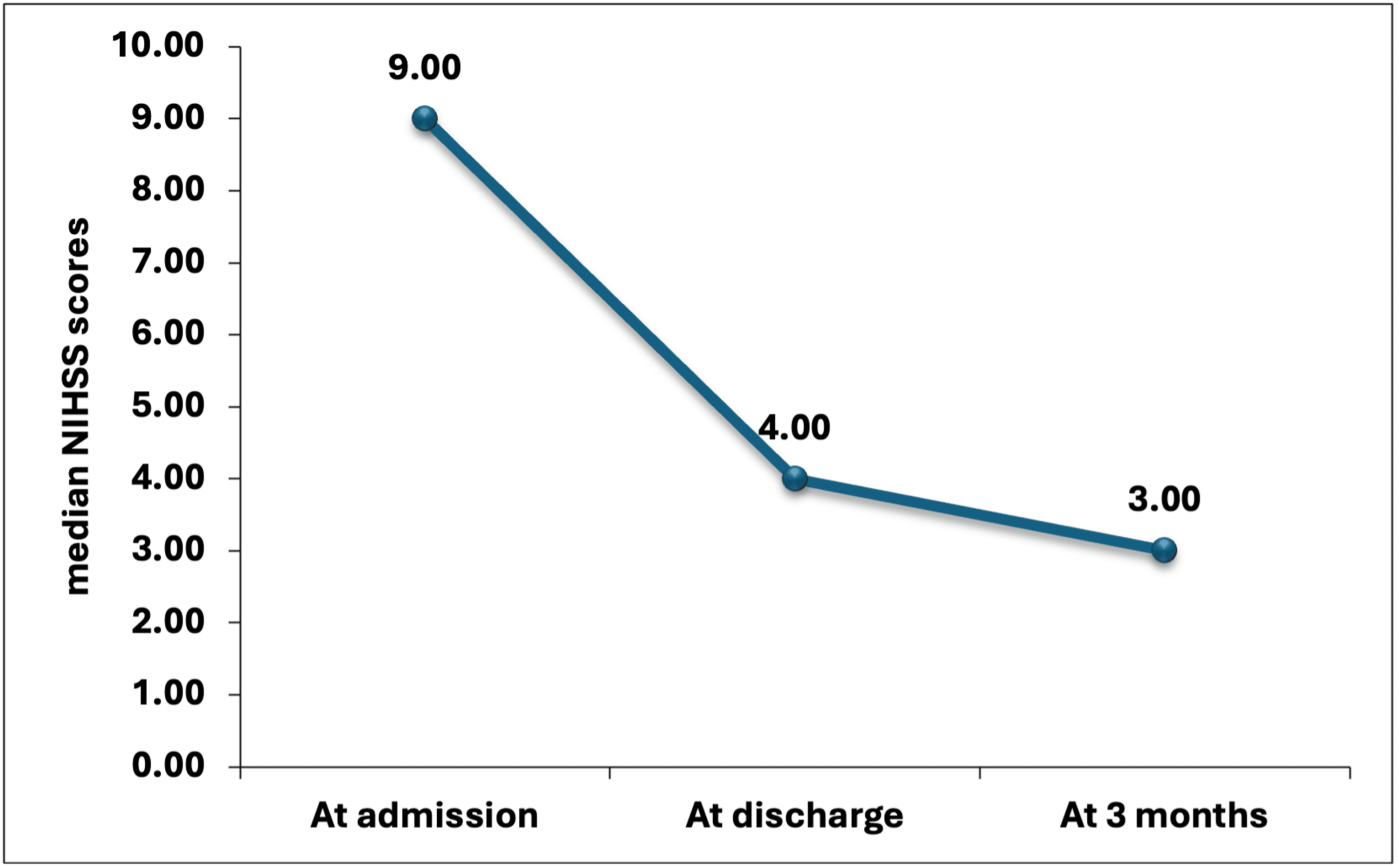
Trends of the NIHSS score *The Wilcoxon signed-rank test compares median NIHSS scores at discharge and at three months against admission values NIHSS: National Institutes of Health Stroke Scale

At the time of admission, 9 was the median NIHSS score. The latter score represents moderate severity of stroke. At the time of discharge, there was a drop in the median value to 4 (p<0.0001). This drop indicates a significant improvement. After three months, a further dip was seen in the median score to 3 (p<0.0001). This drop indicates a continuation in the recovery (Figure 1).

**Figure 2:**
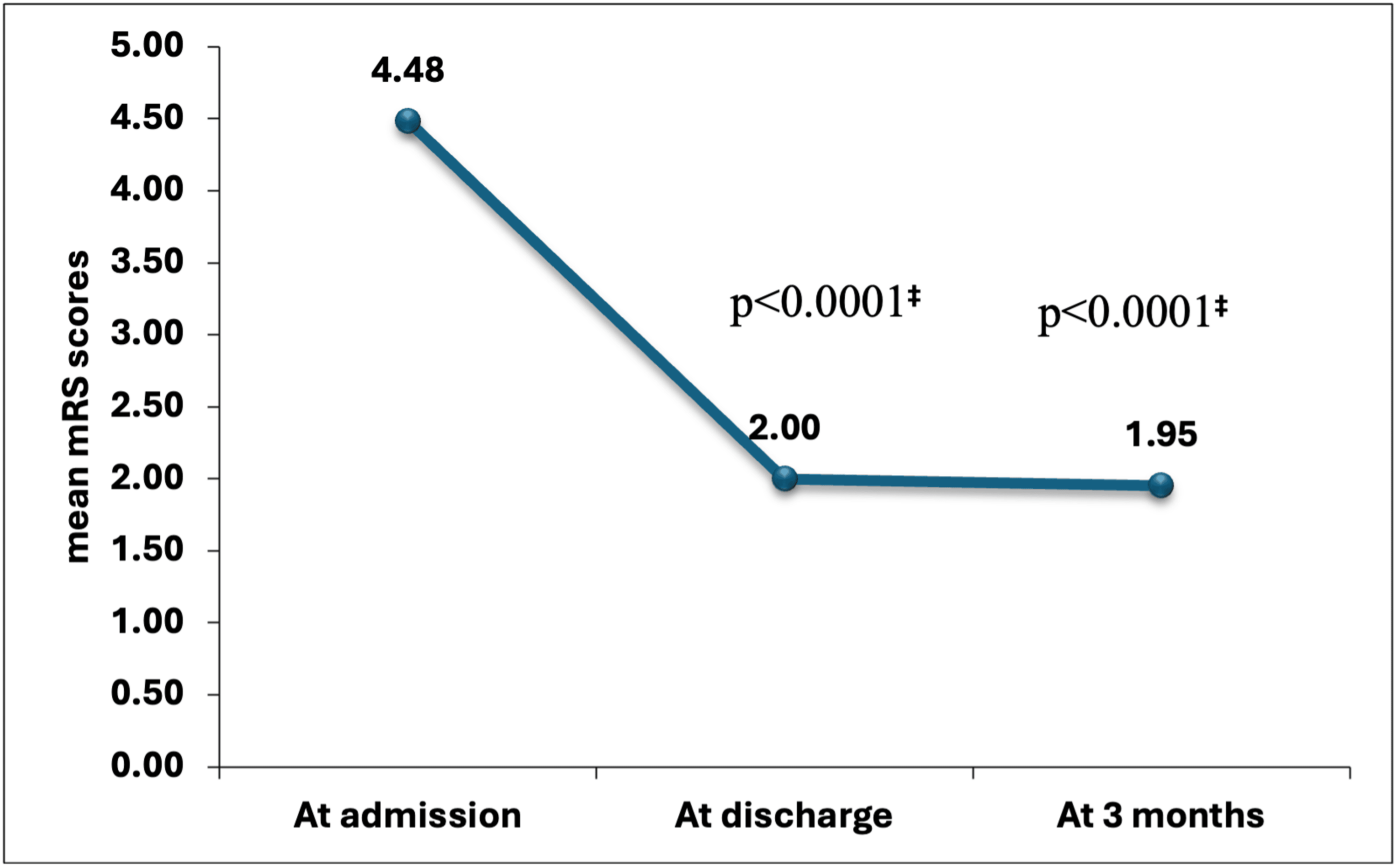
Trends of the modified Rankin scale ^‡^The paired t-test compares median mRS scores at discharge and at three months against admission values mRS: Modified Rankin scale

At the time of admission, 4.48 was the mean value of the mean mRS. This score dropped enormously to 2.00 at the time of discharge (p<0.0001) and to 1.95 after three months (p<0.0001) (Figure 2).

**Figure 3:**
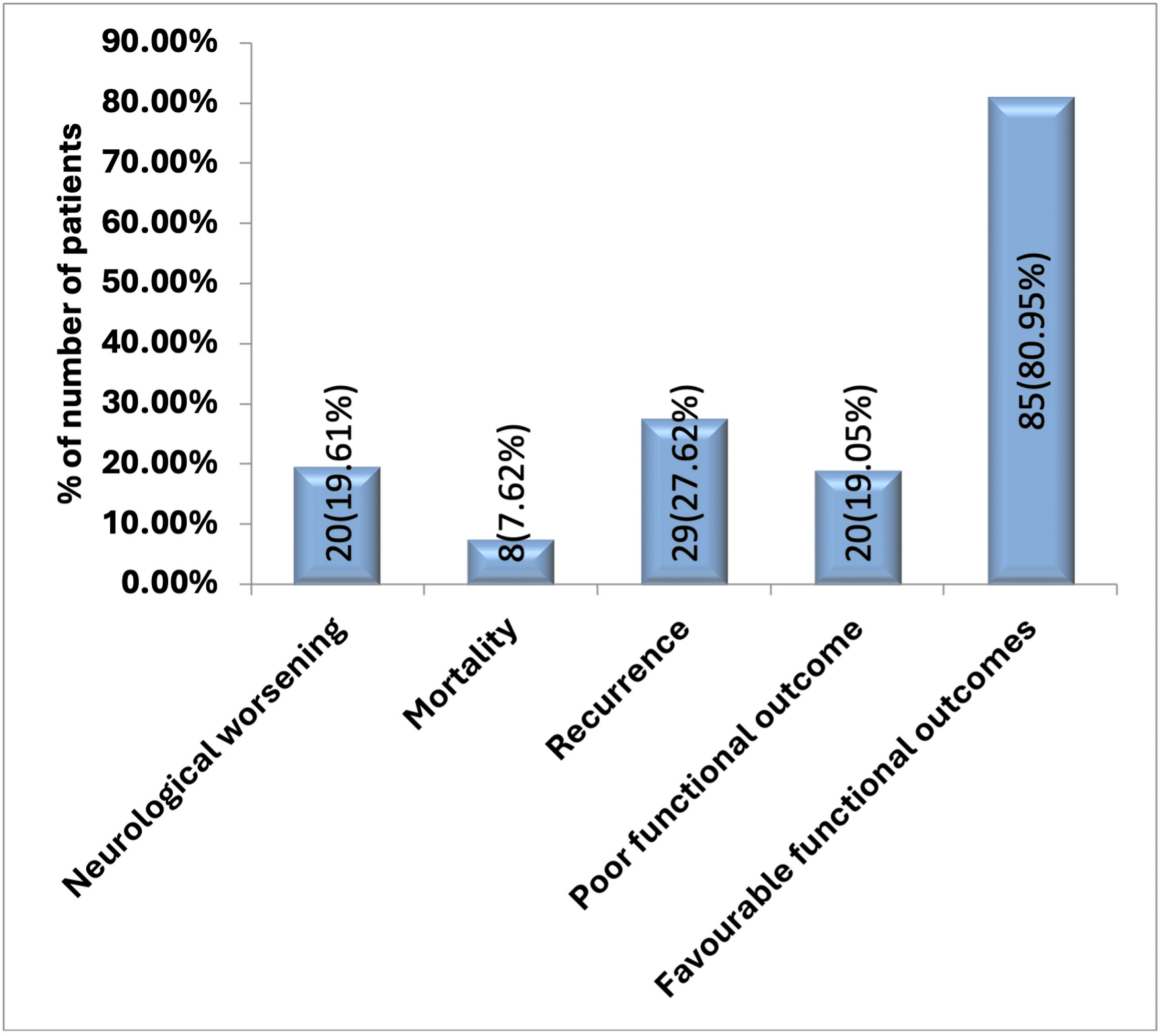
Outcome distribution

Out of the total cases, neurological worsening was seen in 20 cases (19.61%), 29 cases had recurrence (27.62%), and eight cases resulted in mortality (7.62%). A poor functional outcome was seen in 20 patients (19.05%), and favorable functional outcomes were seen in 85 cases (80.95%) (Figure 3).

Patients who had neurological worsening had a significantly higher TyG index in comparison to those who did not have neurological worsening (9.5 ± 0.19 vs. 9.21 ± 0.21, p<.0001). It was also significantly higher in patients having recurrence (9.55 ± 0.18 vs. 9.15 ± 0.15, p<.0001). Patients who had a comparable TyG index showed good functional outcomes when compared with patients having poor outcomes (9.25 ± 0.22 vs. 9.31 ± 0.31, p=0.418) and in non-survivors and survivors (9.27 ± 0.24 vs. 9.12 ± 0.25, p=0.09) (Table 5).

**Table 5:** Association of the TyG index with outcomes of AIS patients TyG: Triglyceride glucose index; AIS: acute ischemic stroke. Three patients died during the hospital stay. So neurological worsening was assessed for 102 patients

Parameters	TyG index (mean +-SD)	Statistical test	Test value	p-value
Neurological worsening		Independent unpaired t-test	t=5.6353	<0.0001
Yes	9.5 ± 0.19			
No	9.21 ± 0.21			
Mortality		Independent unpaired t-test	t=1.6942	0.0932
Survivors (n=97)	9.27 ± 0.24			
Non-survivors (n=8)	9.12 ± 0.25			
Recurrence		Independent unpaired t-test	t=11.5464	<0.0001
Yes (n=29)	9.55 ± 0.18			
No (n=76)	9.15 ± 0.15			
Functional outcomes (mRS)		Independent unpaired t-test	t=1.0095	0.3151
Good (n=85)	9.25 ± 0.22			
Bad (n=20)	9.31 ± 0.31			

At the time of discharge and at three months, the NIHSS score correlated positively with the TyG index in a significant manner with values as r=0.442, p<0.0001 and r=0.398, p<0.0001, respectively. However, there was no notable correlation between the mRS and TyG index (Table 6).

**Table 6:** Correlation of the TyG index with outcome scores Spearman rank correlation coefficient NIHSS: National Institutes of Health Stroke Scale; TyG: triglyceride glucose

Parameters	r-value	p-value
NIHSS		
At admission	-0.073	0.4602
At discharge	0.442	<0.0001
At three months	0.398	<0.0001
mRS		
At admission	0.124	0.207
At discharge	0.045	0.648
At three months	0.07	0.48

## Discussion

The study holds significance as it shows the utility of a newer marker: TyG index for predicting outcomes in AIS patients. At the time of admission, the mean TyG index of the patients with AIS was recorded [9.26 ± 0.2 (range 4-8)]. This was similar to that reported by Nam et al. [20]. As per Wang et al. [25], 129 patients with AIS had a mean TyG index of 9.0. In a study done by Lopez-Jaramillo et al. [26], 8.58 was the value of the mean TyG index. According to Zhang et al. [27], in 676 patients with AIS, the mean TyG index was 8.62.

Deterioration of the neurological structures was determined using the NIHSS score. As per the current study, if at the time of admission, the NIHSS score was high, it resulted in a higher TyG index. At the time of admission, the median NIHSS score was 9. A score of 9 shows moderate stroke severity. At the time of discharge, a significant drop was seen in the median score to 4 (p < 0.0001), this significant drop entails substantial improvement. A further drop in median score was seen to 3 (p < 0.0001), after three months, meaning continued recovery. In our study, data revealed neurological worsening in 20 (19.61%) cases. As per Lee et al. [28], 15 was the median baseline NIHSS score. Early worsening of the neurological structures was seen in 29 (15.8%) cases. According to Toh et al. [29], at baseline, the mean NIHSS score was 14, and after 24 hours, it was 6. In 389 (59.7%) cases, early neurological improvement (ENI) was seen.

A significantly higher TyG index (9.5 ± 0.19 vs. 9.21 ± 0.21, P<.0001) was seen in patients with neurological worsening, in comparison to patients without neurological worsening (n=82). At the time of discharge, a substantial positive correlation was seen in the TyG index with the NIHSS score (r=0.442, p<0.0001). After three months, also a strong positive correlation was seen in the TyG index with the NIHSS score (r=0.398, p<0.0001). This was in accordance with the study by Zhou et al. [19]. In that study, a higher risk of neurological deterioration was seen among patients having a TyG index of Q4 (adjusted OR, 1.26; P = 0.03), at discharge, when compared to those with a TyG index of Q1 [19]. In a study by Lee et al. [28], a correlation was seen between early neurological deterioration (high TyG vs. low TyG index:18.40% vs. 0%, p=0.041) and higher TyG index. Toh et al. [29] reported a substantially lower median TyG index in patients with ENI (8.56 vs. 8.69, p=0.007). A significant association was seen between poorer ENI (OR: 0.68, p=0.004) and TyG index, on multivariate analysis.

To establish functional outcomes, the mRS score was used. At the time of admission, 50 cases (47.62%) presented a score of 5, and 55 cases (52.38%) presented a score of 4, with a mean score of 4.48 ± 0.50. At the time of discharge, the value of the mean score lowered to 2 ± 1.4 (p<.0001), and after three months, it further lowered to 1.95 ± 1.42 (p<.0001). As per the data, poor functional results were seen in 20 (19.05%) cases. Zhou et al. stated poor functional results in 22.4% of patients [19]. As per Lee et al. [28], the TyG index was similar (4.8±0.4 4.9±0.3, p=0.076) in patients with poor results (n = 105), and in patients with good results (n = 78). At 3 months, poor functional results were seen in 103 (56.28%) patients. In a study by Toh et al. [29], 23% of patients had a 0 mRS score at 90 days, 21.4% of patients had a value of 1, and 11.2% of patients had a value of 3. At three months, 308 (44.6%) patients presented with poor functional outcomes (mRS 3-6), after the stroke. Miao et al. [30] performed a study on 3,216 AIS patients; out of them, 748 (23.3%) patients presented with poor functional outcomes.

A similar TyG index (9.31 ± 0.31 vs. 9.25 ± 0.22, p=0.418) was seen in patients having poor functional outcomes and patients not having poor functional outcomes (n=20). At the time of discharge (r=0.045, p=0.648) and after three months (r=0.070, p=0.480), an insignificant association was seen between the TyG index and the mRS score. Zhou et al. also came up with the same results in the studies performed, in which they did a 12-month follow-up [19]. According to Toh et al. [29], there was no substantial difference in the median TyG index among patients with poor and good functional outcomes (8.62 vs. 8.62, p=0.623). A substantial correlation was seen between poor functional outcomes and the TyG index on multivariate analysis (OR: 1.41, p=0.022).

Lee et al. [28], on the other hand, stated that a correlation was seen between three-month poor functional outcome and a higher TyG index (mRS3-6) (high TyG vs. low TyG index: 61.40% vs. 32%, p=0.011), on univariate analysis. At three months, for poor functional outcomes, a high TyG index was an independent indicator, with an odds ratio of 5.22 (p=0.014). Miao et al. [30], in a study, showed that at the time of discharge, there was an increased risk of poor functional outcome, as per the highest fourth quartile of the TyG index with an odds ratio of 1.62 (P=0.002). Heterogeneous populations may result in differences.

In this study, in 29 (27.62%) patients recurrence occurred after three months follow-up. In comparison, Zhou et al. [19] stated that among 16310 participants, 7.3% of patients had a recurrence. 

A substantially higher TyG index (9.55 ± 0.18 vs. 9.15 ± 0.15, P<.0001) was seen in patients having no recurrence, in comparison to those with recurrence. Previous studies were in line with this. In the study by Zhou et al. [19], a correlation was seen between a higher risk of stroke recurrence [adjusted hazard ratio (aHR), 1.32; p = 0.002], and TyG index of Q4 (≥9.21), when compared to patients with a TyG index of Q1 (≤ 8.33), on multivariate analysis.

Yang et al. [21] stated a higher stroke recurrence within one year, in patients with a higher TyG index quartile. Those in the fourth and third (aHR: 2.04) quartile of the TyG index, had a correlation with a higher risk of recurrence of stroke (aHR: 1.86) (P = 0.03). 

Although the mechanism linking the TyG index to stroke recurrence is not yet clearly understood, it may be related to IR. IR is a syndrome associated with a cluster of metabolic disorders. It is linked to obesity, diabetes mellitus, hypertension, hyperlipidemia, inflammation, and atherosclerosis, which are all significant risk factors for stroke recurrence [21]. Moreover, in addition to IR, other factors might have contributed to the recurrence, e.g., continued smoking, poor compliance to antihypertensive, antiplatelet agents, and uncontrolled DM [21].

Even though there is not much clarity on the process of associating stroke recurrence to the TyG index, it is assumed that it may be related to IR (a syndrome related to a cluster of metabolic disorders). It is associated with diabetes mellitus, obesity, hypertension, inflammation, hyperlipidemia, and atherosclerosis (all are important risk factors in stroke recurrence) [21].

Mortality occurred in eight (7.62%) patients, in this study. As per Zhou et al. [19], 1,353 (8.3%) could not survive out of 16310 patients. In the study by Toh et al. [29], 9.1% was the rate of mortality. Miao et al. [30] reported a 3.3% mortality rate. In our study, it was seen that non-survivors had an insignificant correlation with the mean TyG index (9.12 ± 0.25 vs. 9.27 ± 0.24, p=0.09). The factors owing to the latter were probably that the sample was not powered enough to obtain a statistically significant association. Yang et al. [21] also stated that higher mortality risk was seen in patients in the fourth quartile of the TyG index (adjusted hazard ratio of 2.91 (p<0.001)). Toh et al. [29] also found a strong correlation between mortality and the TyG index (OR: 2.12, p=0.001).

According to our study and other studies, the TyG index is valid for finding adverse prognosis like recurrence and neurological worsening [29]. A plausible process could be that IR can cause an increase in prothrombotic responses, chronic proinflammatory cytokines, and endothelial dysfunction (increasing the likelihood of brain damage post-stroke). Resistance to thrombolysis may increase in metabolic syndrome (closely related to IR), due to an increased clot density or impaired fibrinolytic system. Also, people having multiple characteristics of metabolic syndrome may be more resistant to lysis due to denser clot structures. This shows maybe in the future, targeting and identifying IR in patients may help in the prevention of poor response to thrombolysis [29].

Limitations of the study

The results may not be generalized to other settings or populations, due to it being a single-center study. The study findings were also limited due to a small sample size. A lack of a control group of patients (without ischemic stroke) for comparison also limits the ability of the TyG index value contextualization specific to stroke patients. The fasting glucose and triglyceride levels may have been touched by antidiabetic medications or by agents that lower the lipid levels before admission. This makes the TyG index less reliable to assess pre-stroke IR. There is no elaborate data on the long-term outcomes or follow-up period. The aforementioned factors are crucial to understand the prolonged effect of the TyG index on the prognosis of stroke. No assessment was made on cognitive function or quality of life.

## Conclusions

The present study findings revealed a significant association between the TyG index and certain clinical outcomes in AIS patients. Patients with higher TyG indices were more likely to experience stroke recurrence and neurological worsening, suggesting a link between insulin resistance and adverse outcomes in AIS. Specifically, the TyG index was significantly higher in patients with neurological worsening and recurrent strokes. However, no significant association was observed between the TyG index and poor functional outcomes or three-month mortality. These findings highlight the impact of insulin resistance on the pathophysiology and prognosis of AIS. It can be suggested that incorporating the TyG index into clinical practice could aid in risk stratification and early intervention strategies for AIS patients, particularly in addressing modifiable metabolic factors to improve neurological outcomes and reduce stroke recurrence.

## References

[1] Lloyd-Jones D, Adams R, Carnethon M (2009). Heart disease and stroke statistics--2009 update: a report from the American Heart Association Statistics Committee and Stroke Statistics Subcommittee. Circulation.

[2] Rangamani S, Huliyappa D, Kulothungan V (2024). Stroke incidence, mortality, subtypes in rural and urban populations in five geographic areas of India (2018-2019): results from the National Stroke Registry Programme. Lancet Reg Health Southeast Asia.

[3] Kuriakose D, Xiao Z (2020). Pathophysiology and treatment of stroke: present status and future perspectives. Int J Mol Sci.

[4] Ding PF, Zhang HS, Wang J, Gao YY, Mao JN, Hang CH, Li W (2022). Insulin resistance in ischemic stroke: mechanisms and therapeutic approaches. Front Endocrinol (Lausanne).

[5] Fitzgibbons TP, Czech MP (2016). Emerging evidence for beneficial macrophage functions in atherosclerosis and obesity-induced insulin resistance. J Mol Med (Berl).

[6] Gast KB, Smit JW, den Heijer M (2013). Abdominal adiposity largely explains associations between insulin resistance, hyperglycemia and subclinical atherosclerosis: the NEO study. Atherosclerosis.

[7] Slyper AH, Rosenberg H, Kabra A, Weiss MJ, Blech B, Gensler S, Matsumura M (2014). Early atherogenesis and visceral fat in obese adolescents. Int J Obes (Lond).

[8] Vazzana N, Ranalli P, Cuccurullo C, Davì G (2012). Diabetes mellitus and thrombosis. Thromb Res.

[9] Jiao Y, Su Y, Shen J (2022). Evaluation of the long-term prognostic ability of triglyceride-glucose index for elderly acute coronary syndrome patients: a cohort study. Cardiovasc Diabetol.

[10] Hosseini SM (2017). Triglyceride-glucose index simulation. J Clin Basic Res.

[11] Sánchez-García A, Rodríguez-Gutiérrez R, Mancillas-Adame L (2020). Diagnostic accuracy of the triglyceride and glucose index for insulin resistance: a systematic review. Int J Endocrinol.

[12] Selvi NM, Nandhini S, Sakthivadivel V, Lokesh S, Srinivasan AR, Sumathi S (2021). Association of triglyceride-glucose index (TyG index) with HbA1c and insulin resistance in type 2 diabetes mellitus. Maedica (Bucur).

[13] Zhao Q, Zhang TY, Cheng YJ, Ma Y, Xu YK, Yang JQ, Zhou YJ (2021). Triglyceride-glucose index as a surrogate marker of insulin resistance for predicting cardiovascular outcomes in nondiabetic patients with non-ST-segment elevation acute coronary syndrome undergoing percutaneous coronary intervention. J Atheroscler Thromb.

[14] Cho YK, Han KD, Kim HS, Jung CH, Park JY, Lee WJ (2022). Triglyceride-glucose index is a useful marker for predicting future cardiovascular disease and mortality in young Korean adults: a nationwide population-based cohort study. J Lipid Atheroscler.

[15] Irace C, Carallo C, Scavelli FB, De Franceschi MS, Esposito T, Tripolino C, Gnasso A (2013). Markers of insulin resistance and carotid atherosclerosis. A comparison of the homeostasis model assessment and triglyceride glucose index. Int J Clin Pract.

[16] Kim MK, Ahn CW, Kang S, Nam JS, Kim KR, Park JS (2017). Relationship between the triglyceride glucose index and coronary artery calcification in Korean adults. Cardiovasc Diabetol.

[17] Lee EY, Yang HK, Lee J (2016). Triglyceride glucose index, a marker of insulin resistance, is associated with coronary artery stenosis in asymptomatic subjects with type 2 diabetes. Lipids Health Dis.

[18] Lee SB, Ahn CW, Lee BK (2018). Association between triglyceride glucose index and arterial stiffness in Korean adults. Cardiovasc Diabetol.

[19] Zhou Y, Pan Y, Yan H (2020). Triglyceride glucose index and prognosis of patients with ischemic stroke. Front Neurol.

[20] Nam KW, Kwon HM, Lee YS (2021). High triglyceride-glucose index is associated with early recurrent ischemic lesion in acute ischemic stroke. Sci Rep.

[21] Yang X, Wang G, Jing J (2022). Association of triglyceride-glucose index and stroke recurrence among nondiabetic patients with acute ischemic stroke. BMC Neurol.

[22] Aho K, Harmsen P, Hatano S, Marquardsen J, Smirnov VE, Strasser T (1980). Cerebrovascular disease in the community: results of a WHO collaborative study. Bull World Health Organ.

[23] Tian X, Zuo Y, Chen S, Liu Q, Tao B, Wu S, Wang A (2021). Triglyceride-glucose index is associated with the risk of myocardial infarction: an 11-year prospective study in the Kailuan cohort. Cardiovasc Diabetol.

[24] Kwah LK, Diong J (2014). National Institutes of Health Stroke Scale (NIHSS). J Physiother.

[25] Wang J, Tang H, Wang X, Wu J, Gao J, Diao S, Wu Y (2023). Association of triglyceride-glucose index with early neurological deterioration events in patients with acute ischemic stroke. Diabetol Metab Syndr.

[26] Lopez-Jaramillo P, Gomez-Arbelaez D, Martinez-Bello D (2023). Association of the triglyceride glucose index as a measure of insulin resistance with mortality and cardiovascular disease in populations from five continents (PURE study): a prospective cohort study. Lancet Healthy Longev.

[27] Zhang B, Lei H, Ambler G (2023). Association between triglyceride-glucose index and early neurological outcomes after thrombolysis in patients with acute ischemic stroke. J Clin Med.

[28] Lee M, Kim CH, Kim Y (2021). High triglyceride glucose index is associated with poor outcomes in ischemic stroke patients after reperfusion therapy. Cerebrovasc Dis.

[29] Toh EM, Lim AY, Ming C (2022). Association of triglyceride-glucose index with clinical outcomes in patients with acute ischemic stroke receiving intravenous thrombolysis. Sci Rep.

[30] Miao M, Bi Y, Hao L (2023). Triglyceride-glucose index and short-term functional outcome and in-hospital mortality in patients with ischemic stroke. Nutr Metab Cardiovasc Dis.

